# Generation and Validation of *miR-142* Knock Out Mice

**DOI:** 10.1371/journal.pone.0136913

**Published:** 2015-09-01

**Authors:** Amit Shrestha, Gianni Carraro, Elie El Agha, Regina Mukhametshina, Cho-Ming Chao, Albert Rizvanov, Guillermo Barreto, Saverio Bellusci

**Affiliations:** 1 German Center for Lung Research, Excellence Cluster Cardio-Pulmonary System, Universities of Giessen and Marburg Lung Center, Giessen, Hessen, Germany; 2 Lung and Regenerative Medicine Institutes, Department of Medicine, Cedars-Sinai Medical Center, Los Angeles, California; 3 Institute of Fundamental Medicine and Biology, Kazan (Volga Region) Federal University, Kazan, Russian Federation; 4 LOEWE Research Group Lung Cancer Epigenetic Max Plank Institute, Bad Nauheim, Germany; 5 Member of the German Center for Lung Research, Giessen, Germany; 6 Developmental Biology Program, Division of Surgery, Saban Research Institute of Children's Hospital Los Angeles, University of Southern California Keck School of Medicine, Los Angeles, California; Cincinnati Children's Hospital Medical Center, UNITED STATES

## Abstract

*microRNA-142* (*miR-142*) is an important regulator of many biological processes and associated signaling pathways during embryonic development, homeostasis and disease. The *miR-142* hairpin gives rise to the “guide strand” *miR-142-3p* and the sister "passenger" strand *miR-142-5p*. *miR-142-3p* has been shown to play critical, non-redundant functions in the development of the hematopoietic lineage. We have recently reported that *miR-142-3p* is critical for the control of Wnt signaling in the mesenchyme of the developing lung. *miR-142-5p* has been proposed to control adaptive growth in cardiomyocytes postnatally and its increase is associated with extensive apoptosis and cardiac dysfunction in a murine heart failure model. Using homologous recombination, we now report the generation and validation of *miR-142*-null mice. *miR-142*-null mice show a significant decrease in th expression levels of both the *3p* and *5p* isoforms. The expression of *Bzrap1*, a gene immediately flanking *miR-142* is not altered while the expression of a long non-coding RNA embedded within the *miR-142* gene is decreased. *miR-142*-null newborn pups appear normal and are normally represented indicating absence of embryonic lethality. At embryonic day 18.5, *miR-142-*null lungs display increased Wnt signaling associated with the up-regulation of Apc and p300, two previously reported targets of *miR-142-3p* and *-5p*, respectively. Adult *miR-142*-null animals display impaired hematopoietic lineage formation identical to previously reported *miR-142* gene trap knockdown mice. We report, for the first time, the homologous recombination-based *miR-142*-null mice that will be useful for the scientific community working on the diverse biological functions of *miR-142*.

## Introduction

microRNAs (miRNAs) are 22 to 25 nucleotide-long, single stranded RNAs that are processed from hairpin transcripts. The maturation of the hairpin transcript gives rise to the *3p* guide strand and *5p* sister passenger strand, both of which allow the silencing of specific sets of genes through base pairing to a minimal recognition sequence [1,2]. We previously described that *miR-142-3p* is among a few miRs expressed at high level in the lung mesenchyme during early embryonic development [3]. Such restricted expression pattern suggested that *miR-142-3p* could play critical functions in controlling cell lineage formation in the mesenchyme. Using *in-vitro* approaches with embryonic lungs cultured in presence of morpholinos for *miR-142-3p*, we showed that knockdown of *miR-142-3p* leads to arrested proliferation and premature differentiation of smooth muscle progenitor cells. We established that *miR-142-3p* positively regulates Ctnnb1 (ß-catenin) signaling during lung development by targeting *Adenomatous polyposis coli* (*Apc*) mRNA for degradation. Ap*c* negatively regulates Ctnnb1 via direct binding to Ctnnb1 thereby antagonizing the interaction of Ctnnb1 with the transcription factor Tcf. In combination with Axin and Gsk3b, Apc induces ubiquitination and degradation of Ctnnb1 [4]. Using genetic tools, we showed that up-regulation of Ctnnb1 signaling specifically in the mesenchyme via the induced expression of a stable form of Ctnnb1 or the deletion of a copy of *Apc* is sufficient to rescue *miR-142-3p* morpholino-mediated loss-of-function and that *Apc* is a critical target of this miRNA.

Beyond its proposed function in lung development, *miR-142-3p* is one of the highest expressed miRs in various hematopoietic lineages [5,6]. *miR-142-3p* controls neutrophil development in zebrafish [7], orchestrates a network of actin cytoskeleton regulator during megakaryopoiesis [8] and regulates the specification of definitive hemagioblasts during organogenesis [9]. Increased levels of *miR-142-3p* in the serum are associated with recurrence of adenocarcinoma in humans [10]. *miR-142-3p* is a target of Interleukin 6 (IL6) in glioblastoma and it has been proposed that *miR-142-3p* blocks the expression of *IL6*, *Hmga2* and *Sox2* thereby suppressing the stem like properties of glioblastomas [11]. Supporting our *in-vitro* results obtained with *miR-142-3p* during lung development [3], it was reported that *miR-142-3p*, which is up-regulated in human breast cancer stem cells, activates the canonical WNT signaling pathway in a APC-suppression dependent manner [12], resulting in enhanced tumorigenicity. *miR-142-3p* regulates IL6 production upon lipopolysaccharide stimulation in dendritic cells [13]. RNA viruses can also bind *miR-142-3p* to suppress innate immunity promoting neurological disease manifestations [14]. While a significant amount of information is available for *miR142-3p*, our knowledge of *miR-142-5p* is still scarce. In the postnatal heart, *miR-142-5p* targets *p300*, a gene encoding a positive regulator of Wnt signaling and increased expression of *miR-142-5p* is associated with extensive apoptosis and cardiac dysfunction in murine heart failure model [15].

In order to unveil the role of *miR-142* in organogenesis, homeostasis and disease *in-vivo*, we generated and validated the *miR-142-null* mouse. Our validation studies confirm that both *miR-142-3p* and *miR-142-5p* are no longer expressed in these mice. We also show that *miR-142*-null embryonic lungs display increased Wnt signaling confirming the functional role of *miR-142* during lung development. *miR-142*-null adult mice display various hematological abnormalities such as decreased platelet and white blood cell count and increased mean platelet volume indicating that these mice suffer from thrombocytopenia. Similar results were obtained with the *miR-142* knockdown mice developed by Chapnik *et al*., using exogenous gene trap technology [8]. In this paper, we report the generation and validation of a *miR-142*-null mouse line that will be useful for the scientific community working on *miR-142*.

## Materials and Methods

### *miR-142-*null mouse line establishment

The *miR-142-*null mutant mouse line was established at the MCI/ICS (Institut Clinique de la Souris, iCS, Infrastructure Nationale PHENOMIN, 1 rue Laurent Fries, 67404 IIIkrich, France). A MCI proprietary vector containing a floxed neomycin resistance cassette and Protamine-Cre cassette was used (Fig A and B in S1 Fig). The use of Protamine-Cre cassette in the construction vector offers an efficient solution for the auto-excision of the floxed region in the male germ line of mice. In parallel, a 3.3kb fragment (corresponding to the 5’ homology arm) and 2.6kb fragment (corresponding to the 3’ homology arms) were amplified by PCR and sub-cloned into MCI proprietary vector to generate the final targeting construct. The linearized construct was electroporated in C57BL/6N mouse embryonic stem (ES) cells. After selection, targeted clones were identified by PCR using external primers and further confirmed by Southern blotting with 3’ external probe. Two positive recombinant ES clones were injected into BALB/CN blastocysts, and resulting male chimeras were crossed with wild type females. The genotype of mice with germ-line transmission was confirmed using the primers described below. Generated mice were transferred to Mfd Diagnostics (Wendelsheim/Rheinland-Pfalz, Germany), and housed in a SPF environment. Harvesting organs and tissues from wild type and mutant mice following euthanasia using CO_2_ was approved at Justus Liebig University Giessen by the federal authorities for animal research of the Regierungspraesidium Giessen, Hessen, Germany (Approved Protocol No. 452_M).

### PCR Genotyping

*miR-142* heterozygous and null mutant mice were identified by performing PCR on tail genomic DNA using four primers (P1-4). The P2/P3 primers allow detecting the *miR-142* wild type allele (174 bp). The P1/P4 primers allow detecting the presence of the LoxP cassette (105 bp) and the P1/P3 primers allow detecting the deletion of the endogenous *miR-142* gene (294 bp).

(P1) forward: GAA GAA CGA GAT CAG CAG CCT CTG TTC C; (P2) forward: ACG CTA GCA CAG TGT GTG CCC A; (P3) reverse: ACC CAT ATG ATA CAC CAG CCA CGT C; (P4) reverse: GAA GTT ATA CTA GAG CGG CCG TTC AC.

The PCR program consists of a denaturation step at 95°C for 4 min, followed by 34 cycles of denaturation (94°C for 30 s), annealing (62°C for 30 s) and extension steps (72°C for 60 s). The program ends with a completion step at 72°C for 420 s. Each PCR tube contains 2.6 U of Taq polymerase in 5μL of reaction buffer (Qiagen Master Mix), 15 pmol of each primer, 0.5 mM dNTPs and 10 ng of genomic DNA in final volume of 10 μL.

### Murine Peripheral Blood Counts

In the Mfd diagnostics facility, about 250 μL of whole blood was retro-orbitally drawn from 8 weeks-old sex-matched *miR-142*-null and wild type littermates into glass capillary tubes containing 5 μL of 0.5 M EDTA, to prevent coagulation. ADVIVA2120 Hematology System (Siemens Healthcare, Germany) was used to perform complete blood count measurements. Blood count measurement was performed at Justus-Liebig Universitat Klinik fur Kleintiere (Central laboratory for small animals), Giessen Germany.

### miRNA computational analysis

The target prediction and pathway intersection for *miR-142-3p* and *miR-142-5p* were performed using the software mirPath. [16]

### Quantitative Real-time PCR and Statistical Analysis

Freshly isolated embryonic lungs were lysed and total RNA was extracted using miRNeasy Mini or Micro kit (Qiagen, Hilden Germany). 1 μg of RNA was used for cDNA synthesis and RT-PCR for mRNA was carried out using Quantitative Reverse Transcription kit (Qiagen). RT-PCR for miRNA was carried out using Taqman MicroRNA Reverse Transcription kit (Applied Biosystem). In both cases, reactions were assembled following the manufacturer’s recommendations. qPCR was performed using the Light Cycler 480 system (Roche Applied Science). The TaqMan microRNA assay (Applied Biosystem) was used for screening the differential expression of miRNAs whereas SYBR Green (Platinum SYBR Green qPCR SuperMix-UDG Invitrogen) was used for the analysis of mRNA expression. *U6* and *Hprt* (Hypoxanthine phosphporibosyl transferase1) were used as reference genes for normalization of miRNA and mRNA abundance espectively. Results were collected from at least three lung samples and each reaction was run in triplicate. Data were assembled using Graph Pad Prism Software (Graph Pad software, USA) and statistical analyses were performed using Student’s t-test. Data were significant if p<0.05. Primers for *miR142-3p*, *miR142-5p* and U6 were obtained from Applied Biosystems. Primers for Bzrap1: Fwd: 5' AGA GAG CCC TGG GTA CAG C 3', Rev: 5' CCC GAA GCC TAT GTT GAA CT 3'. Primers for LncRNA, Fwd: 5' CTT CCT GAC CCC TGA TAC TTG 3', Rev: 5' CCC ATA TCC TCA CGG ACG 3'. Primers for Apc, Fwd: 5' - CAT GGA CCA GGA CAA GGA CAA AAA CC -3', Rev: 5' - GAA CAC ACA CAG CAG GAC AGA - 3'. Primers for p300, Fwd: 5'-ACA TGA TGC CTC GGA TGA CT -3', Rev: 5'- TAG GGG GCT GTG GCA TAT T -3'. Primers for beta-catenin/Ctnnb1, Fwd: 5'-GCA GCA GCA GTT TGT GGA -3, Rev: 5'-TGT GGA GAG CTC CAG TAC ACC-3.

### Immunofluorescence and *In-situ* hybridization

Tissues were fixed in 4% PFA, gradually dehydrated in ethanol, impregnated with xylene, embedded in paraffin and sectioned into 5 μm slices on poly-L-Lysine-coated slides. Antigen retrieval was performed by treating the sample with Proteinase K for 3 min at 37°C. Slides were blocked twice for 5 min with Dako (DAB Emission +Dual Linksystem HRP, Life Technologies) and then incubated with digoxygenin labeled LNA probes (Exiqon, miRCURY LNA Detection probe, Vedback, Denmark) specific for *miR142-3p* and *miR142-5p*. For immunofluorescence staining the slides were de-paraffinized, blocked with 3% bovine serum albumin (BSA) and 0.4% Triton X-100 (in Tris-buffered saline (TBS) at room temperature (RT) for 1 hour and then incubated with primary antibodies against Apc (#Ab15270, Abcam; 1:200), p-S^552^ß-catenin (#9566, Cell Signaling; 1:200) and p300 (# sc-585, Santa Cruz; 1:200) at 4°C overnight. After incubation with primary antibodies, slides were washed three times in TBST (Tris buffer saline + 0.1% Tween 20) for 5 minutes, incubated with secondary antibodies at RT and washed three times with TBST before being mounted with Prolong Gold Anti-fade Reagent with DAPI (4’,6-diamidino-2-phenylindole;Invitrogen). Photomicrographs of immunofluorescence staining were taken using a Leica DMRA fluorescence microscope with a Leica DFC360 FX camera (Leica, Wetzlar, Germany). Figures were assembled using Adobe Illustrator. The data are representative of at least three lungs from independent experiments.

## Results

### Generation of *miR-142*-null mice

**Fig 1:**
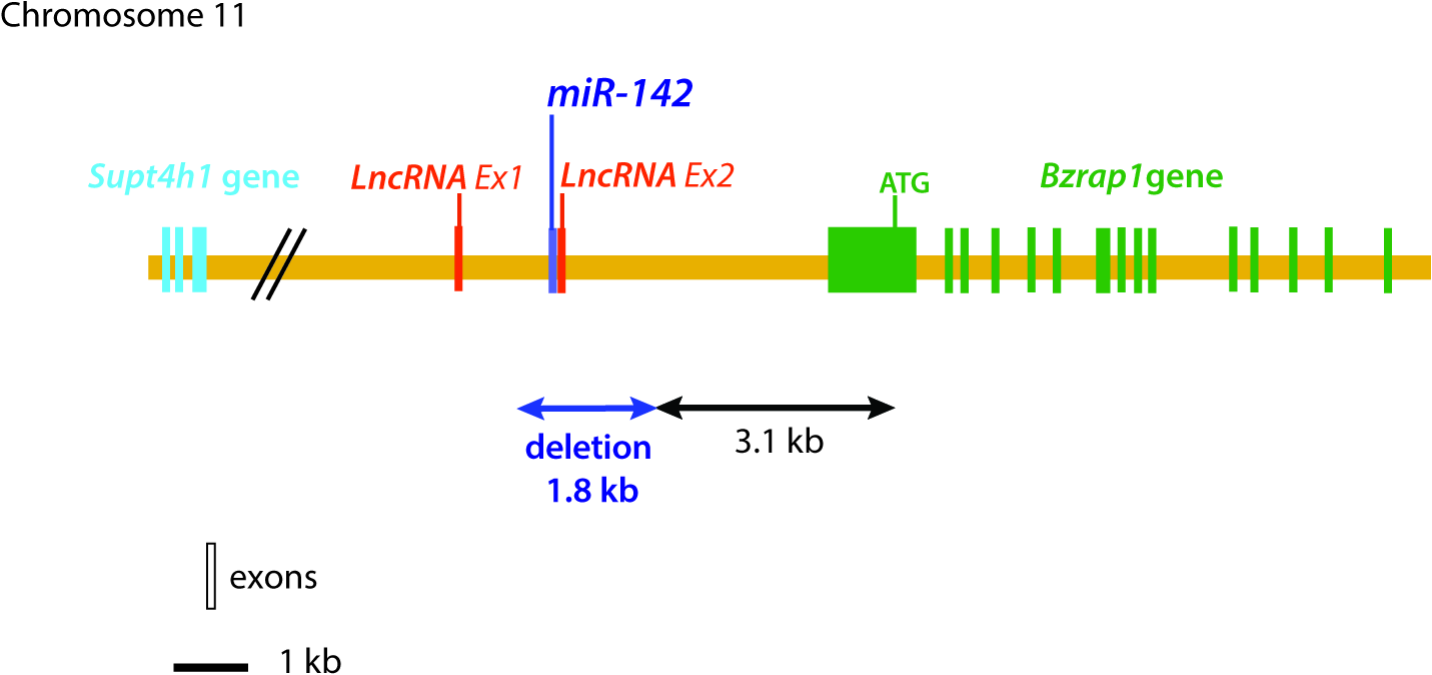
Schematic Representation of the *miR-142* locus in the mouse genome. *miR-142* gene is located in the vicinity of an exon belonging to a LncRNA (A430104N18Rik ENSMUSG00000084796) on chromosome 11. Deletion of *miR-142* led to the deletion of the *LncRNA* exon as well as part of the 5’ UTR of Bzrap1. LncRNA: Long non-coding RNA. Bzrap1: Benzodiazepine receptor (peripheral) associated protein 1.

**Fig 2:**
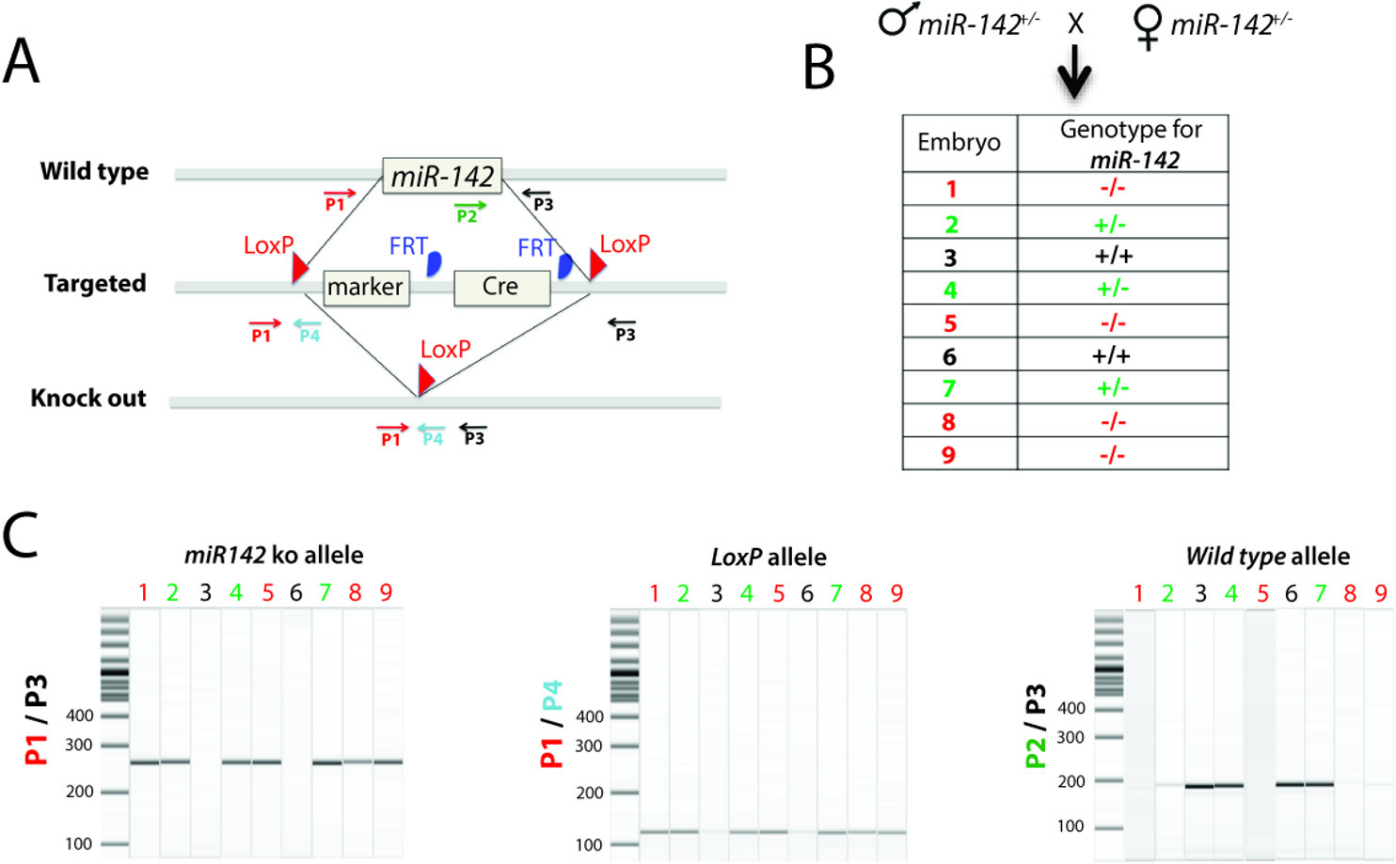
Genotyping Strategy for *miR-142* KO allele. **A)** Schematic diagram showing the WT, targeted and KO alleles for the *miR-142* locus. P1, P2, P3 and P4 represent the primers used to genotype wild type and KO alleles. **B-C)** PCR analysis of the genomic DNA obtained from the embryos at E18.5 from miR-142^+/-^ male and female intercrossing. 254 bp band size for KO allele (Primers used: P1 and P3) and 174 bp band size for WT allele (Primers used: P2 and P3) was determined. A 105 bp band was observed using LoxP specific primers (P1 and P4).

In mice, the *miR-142* gene (ENSMUSG00000065420) is located in chromosome 11 adjacent to the second exon of a long non-coding RNA (A430104N18Rik ENSMUSG00000084796) (Fig 1). The function is still LncRNA is still unknown. *Benzodiazepine receptor (peripheral) associated protein 1* (*Bzrap1*, *ENSMUSG00000034156*) is also another gene found downstream of *miR-142*. The *miR-142* locus also contains many GC-rich repeat regions, which render PCR amplification and screening difficult. The constitutive and complete deletion of the *miR-142* gene was therefore carried out and *miR-142* heterozygous mice were generated. Fig 2B shows the genotyping results of E18.5 embryos arising from crossing *miR-142* heterozygous mice. Fig 2C shows the validation of our primer sets to amplify either the wild type *miR-142* allele (P2/P3), the presence of the *LoxP* site (P1/P4) or the *miR-142*-null allele (P1/P3).

### *miR-142-*null pups are born alive and do not display obvious abnormalities

Litters from *miR-142* heterozygous crosses were genotyped at E12.5, E18.5 and postnatally. Although the expected Mendelian ratio for generating knockout (KO) pups from crossing two hemizygous mice is 25%, this ratio appeared higher when embryos were harvested at E12.5 (39%) and E18.5 (40%) (Table 1). The likely explanation for this observation is the low number of harvested embryos (eighteen and fifteen respectively). Another important conclusion from these data is that *miR-142*-null embryos are not dying *in utero*. In addition, individual monitoring and genotyping of all the neonates born from different litters up to 4 months of age indicate a normal representation of *miR-142*-null mice (20% of the overall postnatal mice (n = 79)). In the *miR-142*-null group, only one 4 month-old mouse died while three animals died in the *miR-142* heterozygous group between the ages of 3 and 4 months. None of the mice died in the control wild type group. Overall, our data indicate very low death rate in the *miR-142*-null group postnatally.

**Table 1:** Number of embryos obtained at different stages of embryonic development.

Embryonic stages	*mir142* ^*+/+*^	*miR142* ^*+/-*^	*miR142* ^*-/-*^	Total embryos
E12.5	3 (17%)	8 (44%)	7 (39%)	18
E18.5	3 (20%)	6 (40%)	6 (40%)	15
Post natal	34 (43%)	29 (36%) #	16 (20%) *	79

# 3 animals died at the age of 2–3 months

* 1 animal died at the age of 4 months

### *miR-142* deletion abolishes the expression of both *miR-142-3p* and *miR-142-5p*

**Fig 3:**
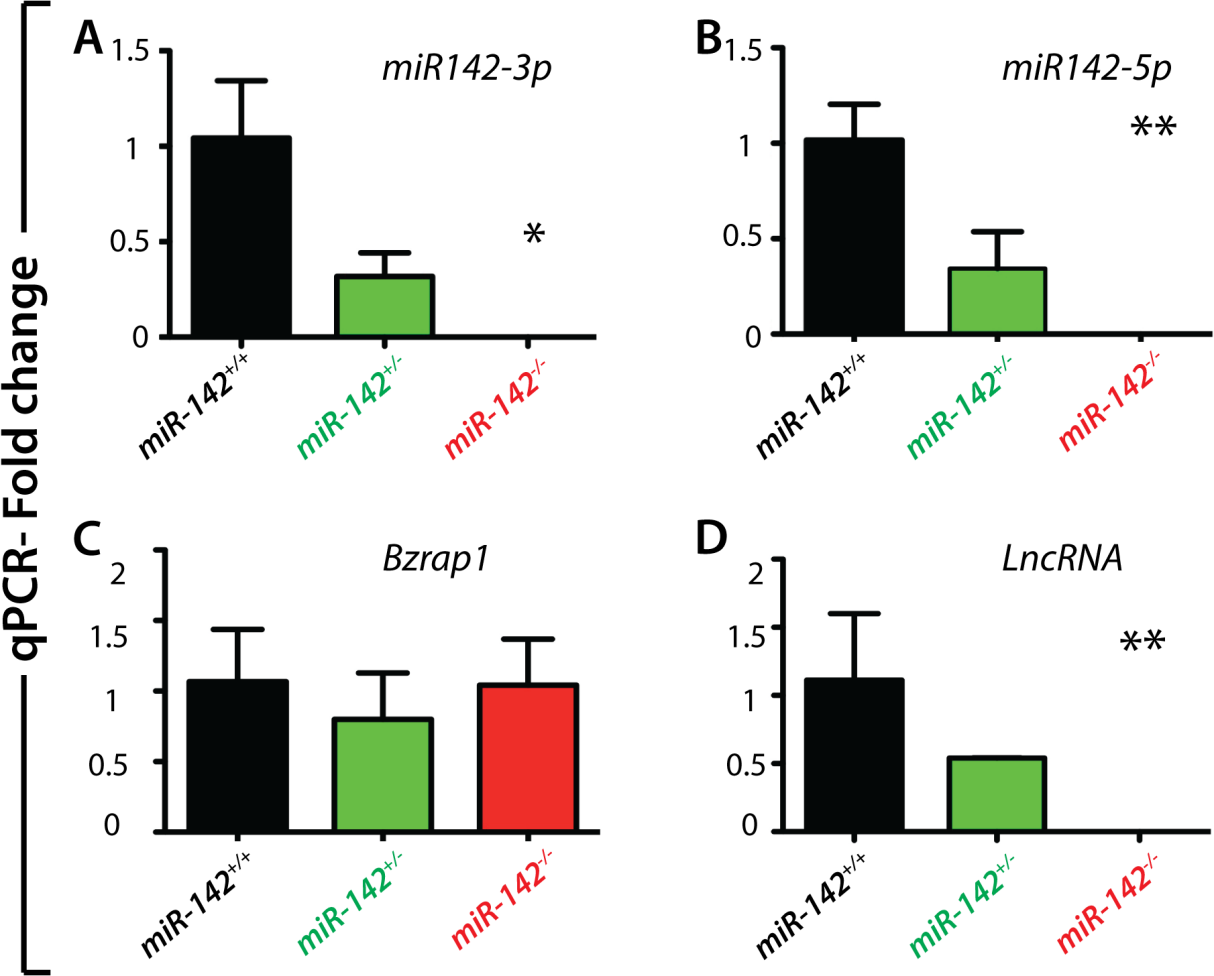
Deletion of *miR-142* successfully abrogates *miR-142-3p* and *miR-142-5p* expression. **A-B)** qPCR analysis on cDNA obtained from E18.5 embryonic lung sample showing that both *miR-142-3p* and *miR-142-5p* are down-regulated in *miR-142* ^*+/-*^ lungs and completely absent in *miR-142*-null lungs as compared to WT samples. **C)** The levels of *Bzrap1* gene, positioned 3.5kb downstream of the *miR-142* gene, remain unchanged as compared to that of wild type littermates shown. **D)** Expression of the LncRNA was completely abolished in *miR-142*-null lungs.

Our experimental approach was designed to carry out the complete deletion of *miR-142*. In the process of deleting *miR-142*, one exon for the *LncRNA*, close to *miR-142*, was also deleted. In addition, part of the 5’ region of *Bzrap1*, a gene encoding a regulator of synaptic transmission, was also deleted. To confirm the successful deletion of *miR-142*, RNA from E18.5 miR-*142* ^*+/+*^, *miR-142* ^*+/-*^ *and miR-142* ^*-/-*^ lungs from littermate embryos (n = 3 for each genotype) were extracted. qPCR analysis showed that the expression of both miRNAs, *miR-142-3p* and *miR-142-5p*, was reduced in *miR-142* heterozygous lungs compared to wild type lungs. In addition, the expression of both isoforms was completely abolished in *miR-142-*null lungs (Fig 3A and 3B). The expression levels of *Bzrap1*, a gene positioned 3.5kb downstream of *miR142*, were unchanged in *miR-142* heterozygous or null lungs compared to wild type littermates (Fig 3C), indicating that the promoter region of *Bzrap1* was not impaired for the expression in the lung. Interestingly, we observed a statistically significant decrease in *Bzrap1* expression in the liver (n = 3 hets vs. 3 ko, p = 0.015) but not in the spleen. In the future, it will therefore be important to validate the changes in *Bzrap1* expression in tissues of interest. As expected, exon 2 of the *LncRNA*, which is located within the deleted region of miR142, was not detected in both *miR-142* heterozygous and null embryos (Fig 3D). It still remains to be demonstrated whether the deletion of this exon leads to the complete loss of function the LncRNA.

**Fig 4:**
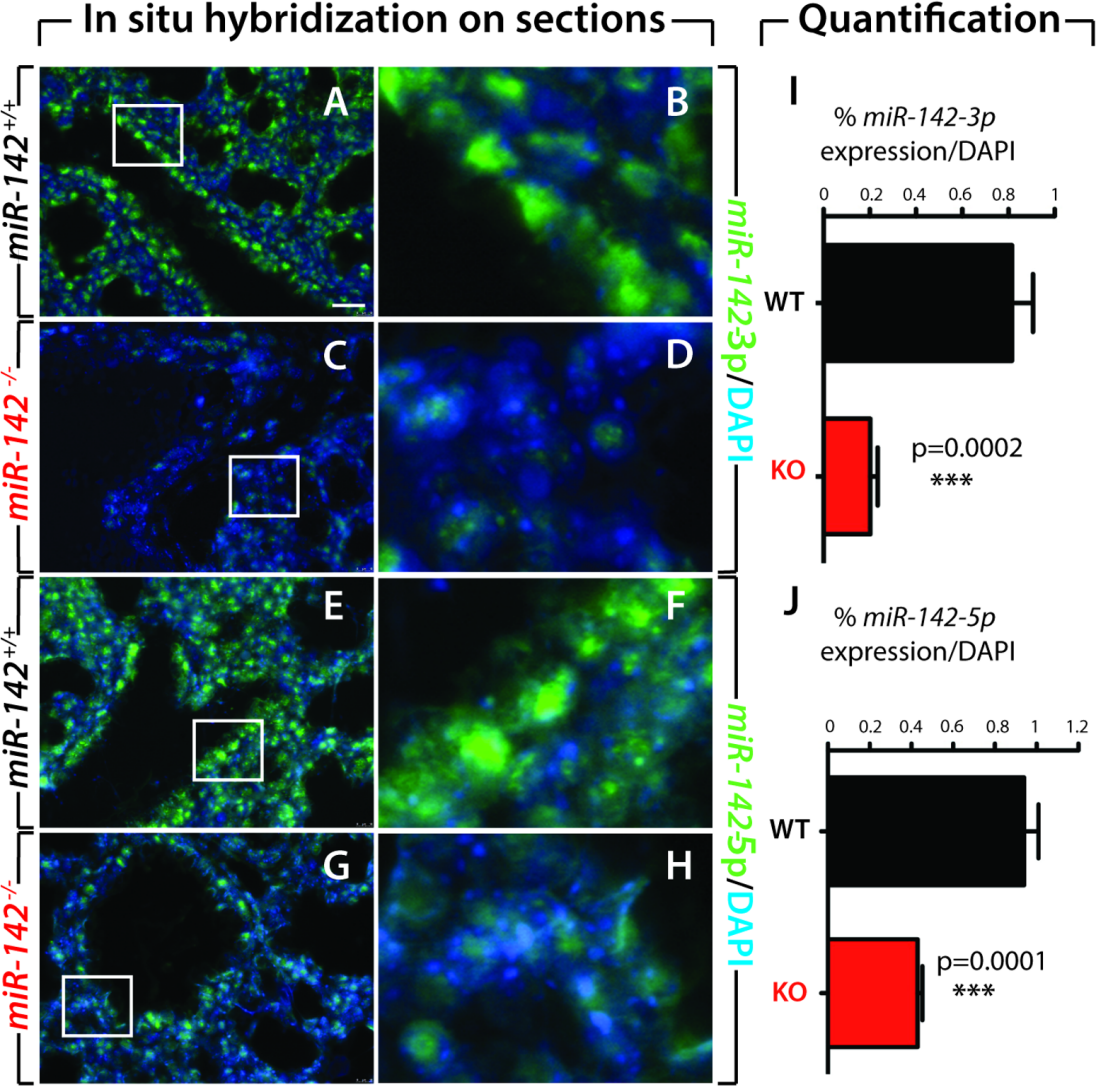
*In situ* hybridization on E18.5 *miR-142* ^*-/-*^ embryonic lung sections. In situ hybridization for *miR-142-3p* (**A-D**) and *miR-142-5p* (**E-H**) showing significant reduction of the corresponding expression levels in *miR-142* ^*-/-*^ compared to WT E18.5 embryonic lung sections. (**I-J**). Quantification of the in situ hybridization signals indicating a strong reduction of both isoforms. Scale bar: (A,C,E,G): 20 μm and (B,D,F,H): 4 μm.

Using specific digoxygenin-labeled probes for these two microRNAs, *in-situ* hybridization showed that *miR-142-3p* and *miR-142-5p* are expressed in both the mesenchyme and the epithelium of the E18.5 lung (Fig 4A, 4B, 4E and 4F). The level of expression of both microRNAs was significantly decreased in *miR142*-null samples compared to WT samples (Fig 4C, 4D, 4G, 4H vs. 4A, 4B, 4E and 4F). Blinded quantification of the expression of the respective isoforms of *miR-142* confirms the very low level of expression of both *miR-142* isoforms (Fig 4I and 4J).

### *Embryonic miR-142*-null lungs display increased Wnt signaling associated with up-regulation of Apc and p300

**Fig 5:**
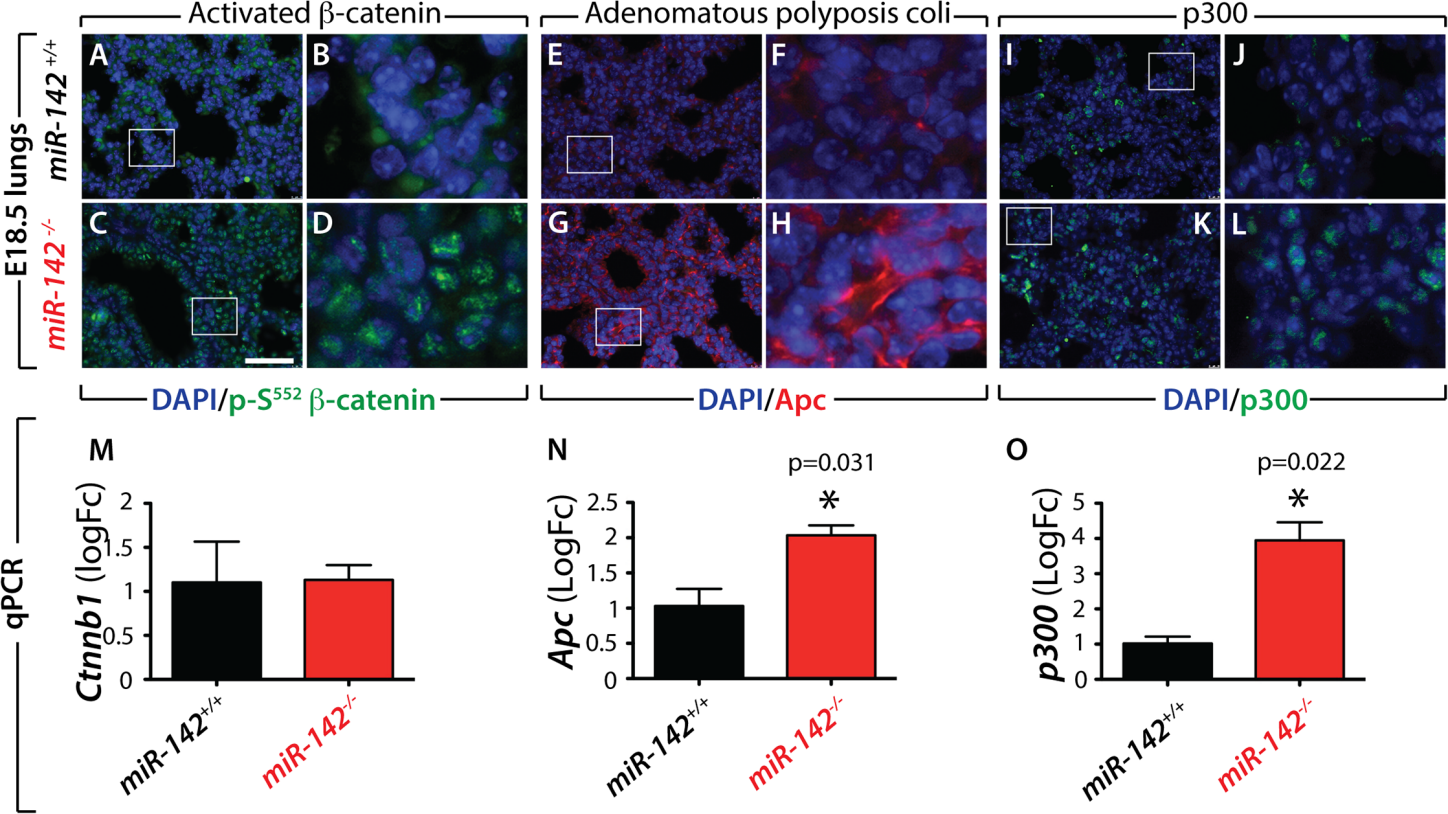
*Increased expression of Apc and p300 is associated with increased Wnt signaling in E18*.*5 miR-142*-null *lungs*. (**A-L)** Immunoflorescence for activated beta-catenin **(A-D)**, Adenomatous polyposis coli (Apc) **(E-H)** and p300 **(I-L)** in wild type (A,B, E,F,I,J) and *miR-142* ko (C,D,G,H,K,L) lungs showing increased Wnt signaling, Apc and p300 expression. **(M-N)** qPCR analysis of beta catenin (*Ctnnb1*), *Apc* and *p300* expression in E18.5 wild type and *miR-142*-null lungs. Scale bar: (A,C,E,G,I,K): 44 μm and (B,D,F,H,J,L): 9 μm.

We previously reported that attenuation of *miR-142-3p* in lung explants grown *in vitro* leads to impaired Wnt signaling in the mesenchyme via the up-regulation of *Apc* [3]. We therefore used immunofluoresence to examine the status of Wnt signaling and Apc in *miR-142*-null versus wild type lungs at E18.5. Surprisingly, our results indicate increased expression of the activated form of Ctnnb1/ß-catenin (p-S552) in the nuclei of *miR-142*-null (Fig 5C and 5D) versus control (Fig 5A and 5B) lungs, suggesting increased Wnt signaling upon loss of *miR-142*. However, and in agreement with our previous report [3], *miR-142*-null lungs display increased expression of Apc (Fig 5G and 5H vs. 5E and 5F). Next, we investigated the expression of *p300*, a previously validated target of *miR-142-5p* [15]. p300 is a positive regulator of Ctnnb1 that synergistically activates Ctnnb1/TCF transcription [17]. Our results indicate increased expression of p300 in *miR-142*-null versus wild type lungs (Fig 5K and 5L vs. 5I and 5J). The respective increase in *Apc* and *p300* in *miR-142-*null lungs was also validated at the mRNA level (Fig 5N and 5O). *Ctnnb1* mRNA expression is not changed between *miR-142*-null and control lungs (Fig 5M).

### *miR-142*-null mice display a wide range of hematological disorder

**Fig 6:**
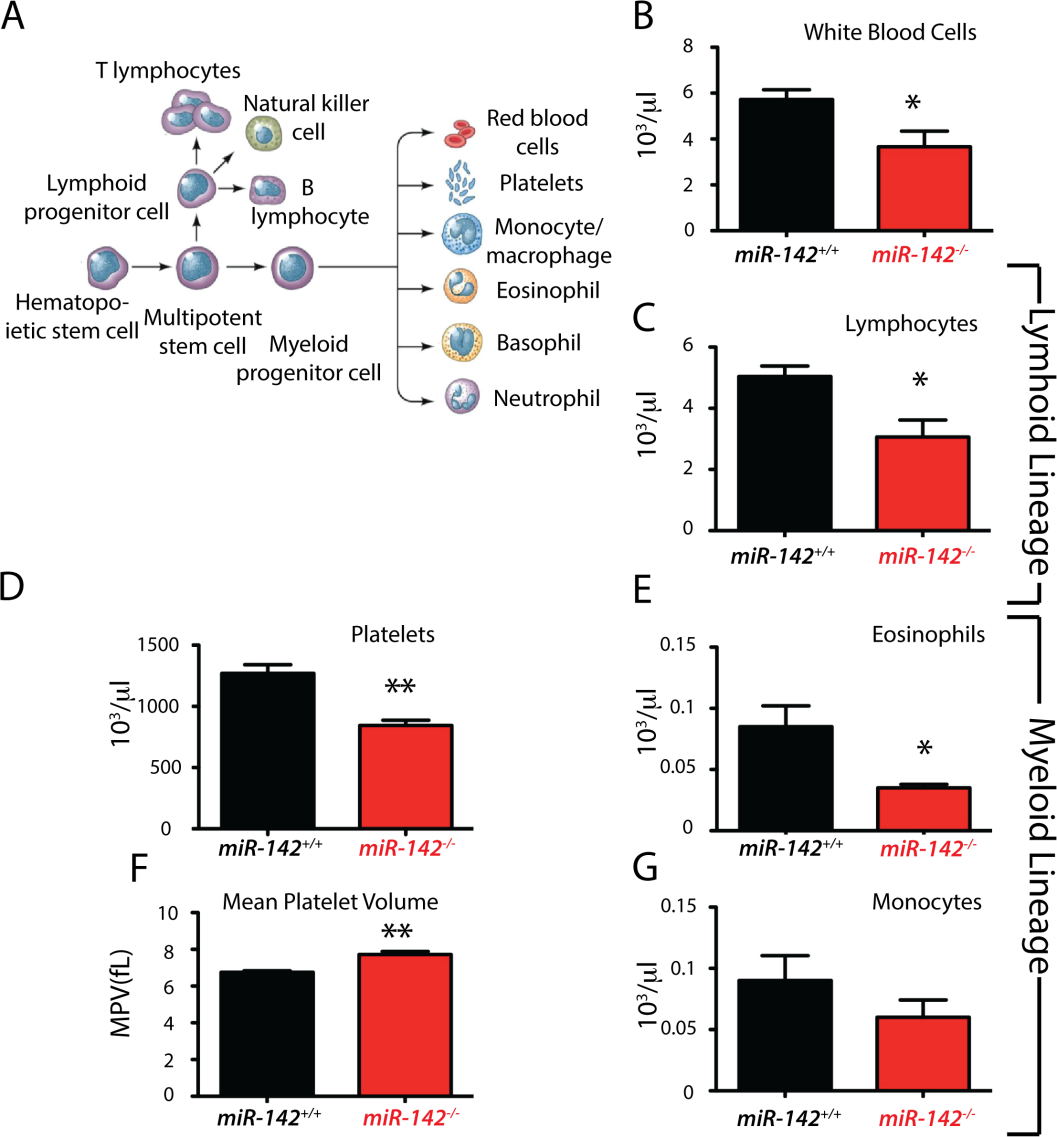
*miR-142* KO mice display hematological abnormalities. **(A**) Schematic representation of hematopoietic stem cells differentiated into different blood cell types. Peripheral blood cell count in 8 week old *miR-142* ^*-/-*^ mice (n = 3) showing a significant decrease in circulating white blood cells (**B**), lymphocytes **(C**), platelets (**D**), eosinophils (**E**) and monocytes (**G**) in addition to an increase in mean platelet volume (**F**).

*miR-142* is highly expressed in hematopoietic cells belonging to both the myeloid and lymphoid lineages. Using an exogenous gene trap technology, Chapnik and colleagues recently reported the phenotypic analysis of *miR-142* knockdown mice. Their mutant mice displayed an array of hematological defects [8]. In order to validate our mouse model, we performed total blood count on 8 week-old *miR-142* ^*+/+*^ and *miR-142* ^*-/-*^ mice (n = 3 for each genotype). Our results indicate a significant decrease in the number of white blood cells, lymphocytes, eosinophils, monocytes and platelets in *miR-142*-null vs. wild type animals (Fig 6B–6E and 6G). A significant increase in mean platelet volume was also observed (Fig 6D and 6F).

## Discussion

We are the first group to report the generation and validation of classical *miR-142-*null mice. We show that both isoforms of *miR-142*, the *3p* and *5p*, are no longer expressed in *miR-142-*null mice. The expression of *Bzrap1*, a gene immediately flanking *miR-142*, is not altered while the expression of a LncRNA embedded within the *miR-142* gene is abolished. E18.5 *miR-142-*null lungs display increased Wnt signaling associated with the up-regulation of *Apc* and *p300*, two previously reported targets of *miR-142-3p* and *-5p*, respectively. *miR-142*-null pups are born alive and are normally represented indicating absence of embryonic lethality. Adult *miR-142*-null animals are viable and display impaired hematopoietic lineage formation. This novel, homologous recombination-based, *miR-142*-null mouse line will be useful for the scientific community.

A *miR-142* gene-trap allele was recently created by insertion of an exogenous gene trap sequence 50 bp upstream of the murine pre-*miR-142* [8]. In these mice, the expression of both *3p* and *5p* was reduced to very low levels but not completely abolished. The authors describe that at E14.5, embryos homozygous for the mutant allele display a normal Mendelian distribution. However, one third of the postnatal homozygous mutant mice died within 3 weeks. Our results support these observations with the exception of the lack of perinatal death. Even though this remains to be shown, perinatal death may be associated with heart failure due to the absence of adaptive growth of the cardiomyocytes [15]. Similarly to the homozygous *miR-142* gene-trap mice, our *miR-142*-null mice also display decreased white blood cells, decreased platelets and increased mean platelet volume. However, we did not observe the decrease in red blood cell numbers as well as the increased level of basophils and reticulocytes (data not shown) initially reported in the homozygous *miR-142* gene-trap mice. The basis for such differences is not known so far and could be linked to the genetic background.

**Fig 7:**
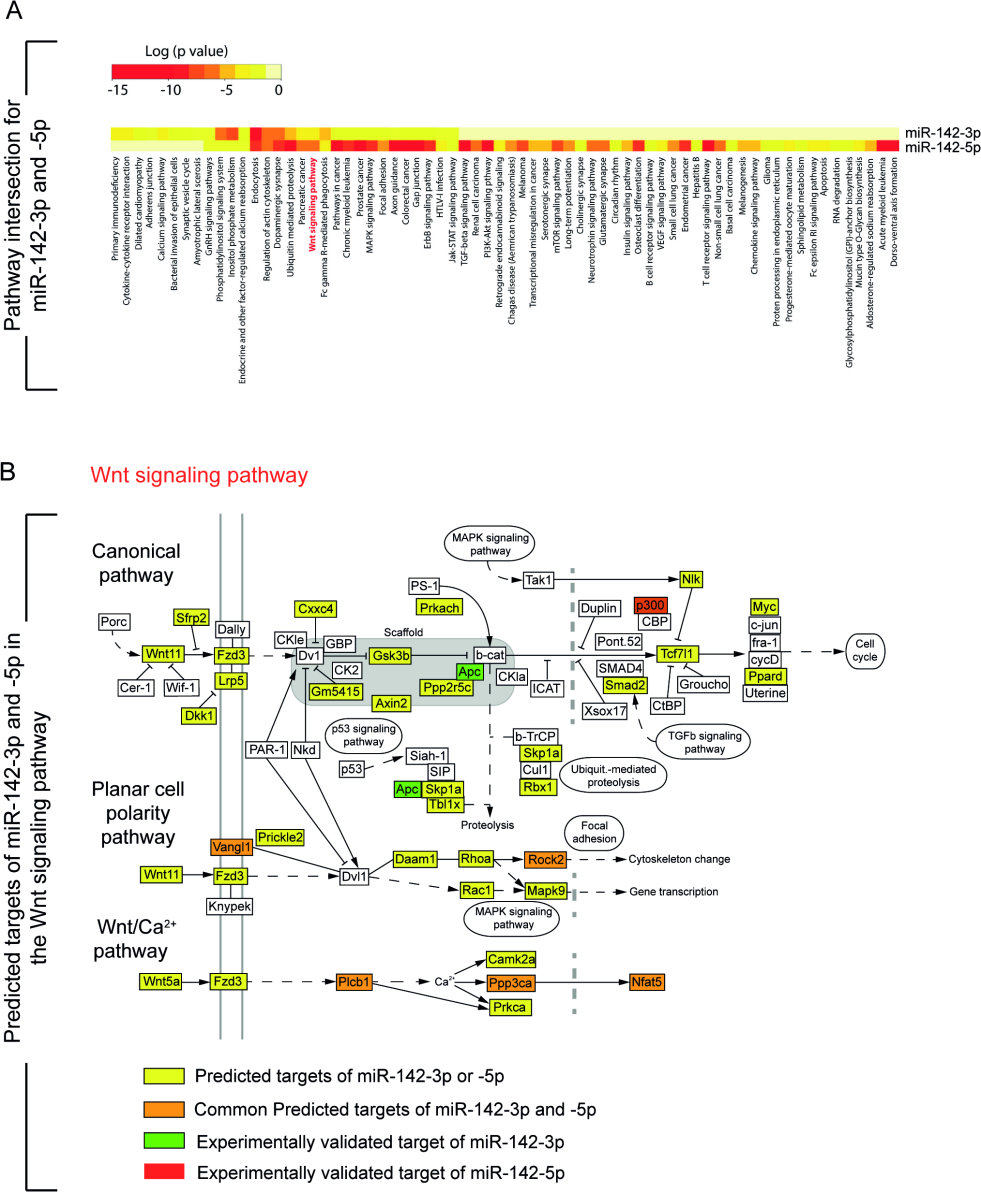
Predicted targets of *miR-142-3p* and *miR-142-5p* using Diana-MicroT and Target Scan software prediction tools. **(A)** Pathway intersection between *miR-142-3p* and *miR-142-5p*. **(B)** Predicted targets of *miR-142-3p* and *miR-142-5p* in the Wnt signaling pathway. Orange boxes: common predicted targets for both isoforms; yellow boxes: predicted targets for both isoforms; green boxes: experimentally validated target of *miR-142-3p*; red box: experimentally validated target of *miR-142-5p*.

In order to predict the potential defects observed in *miR-142*-null animals, the predicted targets for *miR-142-3p* and *5p* were characterized using Diana-MicroT and Targetscan (Fig 7A). Among the processes and pathways potentially affected, we identified endocytosis, regulation of actin cytoskeleton, pathways in cancer, TGFß signaling and Wnt signaling,

As the *miR-142* conventional KO leads to the deletion of both *miR-142-3p* and *miR-142-5p*, it might be challenging to tease out the activity of each isoform. Others and we have previously reported that *Apc* is a direct target of *miR-142-3p* [3,12]. Fig 7B describes the common targets (in orange) for *miR-142-3p* and *5p* as well as unique targets (yellow) for each isoform in regard to the Wnt pathway. Reduction in *Apc* leads to the activation of canonical Wnt signaling by preventing the recruitment of Ctnnb1 to the degradation complex. Increased *Apc* expression upon *miR-142-3p* silencing causes down-regulation of Wnt signaling [3]. On the other hand Sharma et al. found that *p300* is a target of *miR-142-5p*. Interestingly, this target was not identified as a main putative target through *in-silico* analysis and demonstrates the importance of identifying and confirming interacting partners through pull-down assays followed by gene arrays [3] or next generation sequencing. A mutual inhibitory loop between *p300* and *miR-142-5p* has also been described and under mechanical stress, p300 is accumulated in the cardiac cells, which results in the down-regulation of *miR-142-5p*. The loss of *miR-142-5p* expression activates genes required for myocyte survival and function [15]. p300 is a positive regulator of Ctnnb1, which synergistically activates Ctnnb1/TCF transcription [17]. It is therefore not surprising to find that the loss of *miR-142* leads to increased expression of both Apc and p300 (Fig 5). As Apc is a negative regulator and p300 is a positive regulator of Wnt signaling, we expect that upon deletion of *miR-142*, the balance between Apc and p300 will lead to either no change in Wnt signaling, an increase or a decrease in Wnt signaling. In the E18.5 lung, our results indicate that the Apc/p300 balance favors Wnt signaling. The regulation of the relative expression of both isoforms in specific tissues over time is still unknown. In the embryonic lung at E18.5, both the -*5p* and -*3p* isoforms are normally co-expressed in the epithelium and mesenchyme while *-3p* appears to be more abundantly expressed in the mesenchyme at E12.5 [3]. In the hematopoietic system, *-3p* is the predominant isoform expressed. In the heart, it appears that *-5p* is the major isoform. Our mouse model will allow determining the overall contribution of *-3p* and *-5p* to Wnt signaling in different tissues and at different developmental stages.

Another aspect related to the reported *miR-142*-null mouse is the deletion of the LncRNA together with the *miR-142* gene. LncRNAs contribute also to gene regulation [18] and in the future, the generation of new mouse models allowing the tissue-specific rescue of *miR142-3p/5p* expression in the context of the *miR-142* KO animals will allow determining the role of this *LncRNA*. Interestingly, the expression of this *LncRNA* has not been investigated in the *miR-142* gene-trap line. More recently, a conditional KO for *miR-142* was generated by deleting 900 bp of the genomic sequence that encompasses the *miR-142* precursor in the germ line [19]. The authors reported that *miR-142*-null mice are born at the expected Mendelian ratio and appear healthy and fertile with no apparent internal organ defects, similarly to the findings described in this report. They also described a highly penetrant splenomegaly that is also observed in our *miR142*-null mice (data not shown).

In conclusion, we report the generation and validation of a novel homologous recombination-based *miR-142* KO mouse model. Interestingly, these mice display differences as compared to the previously described gene-trap model, which will need to be analyzed further to validate the proposed function of *miR-142* in hematopoiesis. Together with the recently reported conditional *miR-142* KO, our mouse model will be the first generation of tools designed to decipher the role of *miR-142* in development and disease.

## Supporting Information

S1 Fig Schematic representation of *mir-142* targeting vector. (A) Map of *miR-142* targeting vector plasmid.(B) Deletion of *miR-142* locus in the genome.
(TIF)
