# Supplementary figures and images for: Generation and Validation of miR-142 Knock Out Mice
Source: PLoS One. 2015 Sep 1;10(9):e0136913. doi: 10.1371/journal.pone.0136913 (PMC4556616; doi:10.1371/journal.pone.0136913)

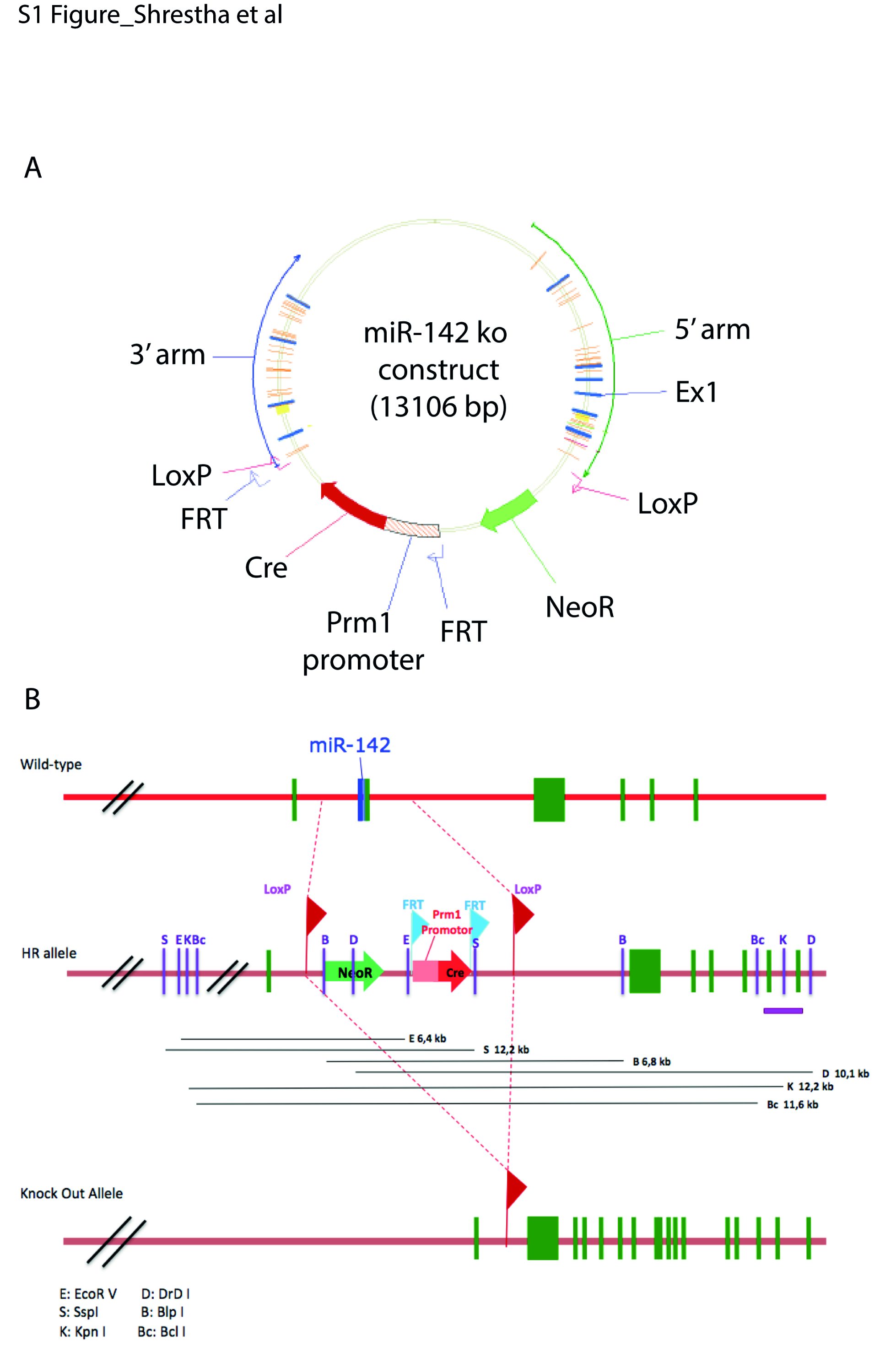

Supplement: S1 Fig — (A) Map of miR-142 targeting vector plasmid.(B) Deletion of miR-142 locus in the genome. (TIF) [file pone.0136913.s001.tif]
